# Host tree phenology affects vascular epiphytes at the physiological, demographic and community level

**DOI:** 10.1093/aobpla/plu073

**Published:** 2014-11-11

**Authors:** Helena J. R. Einzmann, Joachim Beyschlag, Florian Hofhansl, Wolfgang Wanek, Gerhard Zotz

**Affiliations:** 1 Department of Biology and Environmental Sciences, Carl von Ossietzky University of Oldenburg, Carl-von-Ossietzky-Straße 9-11, D-26111 Oldenburg, Germany; 2 Department of Microbiology and Ecosystem Science, University of Vienna, Althanstrasse 14, A-1090 Vienna, Austria; 3 Smithsonian Tropical Research Institute, Apartado Postal 0843-03092, Balboa, Ancon, Panamá, República de Panamá

**Keywords:** Community assembly, Crassulacean acid metabolism (CAM), diversity, microclimate, specific leaf area, water-use efficiency.

## Abstract

The processes that govern diverse tropical plant communities have rarely been studied in life forms other than trees. Structurally dependent vascular epiphytes, a major part of tropical biodiversity, grow in a three-dimensional matrix defined by their hosts, but trees differ in their architecture, bark structure/chemistry and leaf phenology. We hypothesized that the resulting seasonal differences in microclimatic conditions in evergreen vs. deciduous trees would affect epiphytes at different levels, from organ physiology to community structure. We studied the influence of tree leaf phenology on vascular epiphytes on the Island of Barro Colorado, Panama. Five tree species were selected, which were deciduous, semi-deciduous or evergreen. The crowns of drought-deciduous trees, characterized by sunnier and drier microclimates, hosted fewer individuals and less diverse epiphyte assemblages. Differences were also observed at a functional level, e.g. epiphyte assemblages in deciduous trees had larger proportions of Crassulacean acid metabolism species and individuals. At the population level a drier microclimate was associated with lower individual growth and survival in a xerophytic fern. Some species also showed, as expected, lower specific leaf area and higher δ^13^C values when growing in deciduous trees compared with evergreen trees. As hypothesized, host tree leaf phenology influences vascular epiphytes at different levels. Our results suggest a cascading effect of tree composition and associated differences in tree phenology on the diversity and functioning of epiphyte communities in tropical lowland forests.

## Abstract (precis)

The hosts of vascular epiphytes differ in many ways, not least in leaf phenology. We hypothesized that differences in microclimatic conditions in evergreen vs. deciduous trees would affect epiphytes at various levels, from organ physiology to community structure. Indeed, deciduous tree species hosted less abundant and species-poorer epiphyte assemblages. Physiologically, epiphyte assemblages differed in the proportion of CAM species and individuals, and in SLA and δ^13^C values. Effects were also detectable at a demographic level, i.e. in growth and survival rates. Although not all studied epiphyte species showed these effects, the data generally support our basic hypothesis.

## Introduction

Tropical forests are characterized by an unmatched diversity of organismal forms. More than 300 tree species may be found in a single hectare of forest (Balslev *et al.* 1998); and a single tree may in the extreme case be inhabited by almost 200 species of vascular epiphytes (Catchpole and Kirkpatrick 2010). Currently, studies on processes that shape tropical plant communities show a very strong bias towards a single life form, i.e. trees (e.g. Kraft and Ackerly 2010; Metz 2012; Baldeck *et al.* 2013; Brown *et al.* 2013). However, other life forms, e.g. understorey herbs and shrubs, lianas and epiphytes, can make up a substantial proportion of local plant diversity (Gentry and Dodson 1987). Indeed, in some humid montane forests, epiphytes alone may account for a similar fraction of local vascular plant diversity as all other plant life forms combined (Kelly *et al.* 2004).

It is hardly conceivable that vascular epiphyte assemblages made up of structurally dependent, mostly herbaceous plants arranged in a three-dimensional matrix of available substrate supplied by trees are structured by the same biotic and abiotic factors as their hosts. For example, interspecific competition and the impact of pathogens and herbivores, which are very important for trees, seem to be rather irrelevant for epiphytes (Zotz and Hietz 2001). One important biotic factor affecting community assembly of these structurally dependent plants could be host tree identity itself. Arguably, narrow host tree specificity is found only in exceptional cases, whereas a certain degree of host preference is not uncommon (ter Stege and Cornelissen 1989; Laube and Zotz, 2006; Martinez-Melendez *et al.*, 2008). Mechanistically, such preferences are probably related to differences in tree architecture, bark structure and chemistry or leaf phenology of the host. Furthermore, there is also stratification within individual trees (Freiberg 1996; Freiberg, 1999; Krömer *et al.*, 2007). ter Stege and Cornelissen (1989), for example, found that epiphyte assemblages on lower canopy branches (Johansson zone III; Johansson 1974) differed from those on middle and outer canopy branches (Johansson zones IV and V) in a lowland rainforest in Guyana. Several studies have investigated the impact of a variety of tree characteristics on epiphyte distribution, such as tree size (Zotz and Vollrath 2003), branch diameter (Zimmerman and Olmsted 1992) or bark water-holding capacity (WHC, Callaway *et al.* 2002).

Differing tree characteristics create a variety of microclimates within a tree crown that should directly impact epiphyte distributions (Pittendrigh 1948; Benzing, 1995). The changing light regime along the vertical axis of the canopy is one important factor. Lower and more central crown parts are more humid than the outer portions and exposed parts within a tree are usually the driest (e.g. Freiberg 1997; Wagner *et al.* 2013). Stem diameter, tree height and other architectural features of tree species seem to influence stem flow (Hölscher *et al.* 2003), and the stratification of the crown structure influences the amount and the chemical composition of rainwater reaching the epiphytes (Hofhansl *et al.* 2012). However, gradients are not stable in time but are affected by phenological changes of the tree usually associated with wet and dry seasons. Seasonality may be an important factor for the establishment of epiphytes; especially when the host tree is deciduous (Zotz and Winter 1994). Indeed, it has been found that differing microenvironments in evergreen and deciduous trees can lead to significant differences in epiphyte cover (Cardelus 2007). The situation is complex, however, because other traits, e.g. bark characteristics, can aggravate or, alternatively, counteract the possible effects of phenology since epiphyte growth is significantly higher in trees with bark with high WHC (Callaway *et al.* 2002).

The work presented here was initiated to understand the effect of tree leaf phenology on vascular epiphytes at different levels, from leaf traits of individual epiphytes and demographic parameters to community composition, both at a taxonomic and a functional level. To this end, we documented the differences in environmental conditions in the crowns of five tree species on Barro Colorado Island (BCI), which were deciduous, semi-deciduous and evergreen. We hypothesized that:

- deciduous trees host less diverse epiphyte communities,
- traits usually interpreted as adaptation to drought such as Crassulacean acid metabolism (CAM) are more prevalent in epiphyte communities in deciduous trees,
- individual growth is lower in epiphyte populations growing in deciduous trees,
- trait differences associated with drought are also detected at an intraspecific level, thus epiphytes growing in deciduous trees have different trait values such as lower specific leaf area (SLA) and higher δ^13^C values compared with conspecifics growing in evergreen trees.

## Methods

### Study site and survey

The study was conducted on BCI, Republic of Panama. This biological reserve in the Gatun Lake of the Panama Canal, which is administered by the Smithsonian Tropical Research Institute (STRI), is covered by a semi-deciduous lowland forest with varying canopy heights of up to 50 m (Leigh *et al.* 2004). The annual precipitation averages 2600 mm with a pronounced dry season from January through April. During these 4 months, both total rainfall and its frequency are strongly reduced. Rainless periods regularly expand to around 10 days, the maximum rainless period on record being 31 days (based on data from 2006 to 2012 measured by the Physical Monitoring Program of STRI—http://biogeodb.stri.si.edu/physical_monitoring/research/barrocolorado, 10 November 2014).

**Figure 1.**
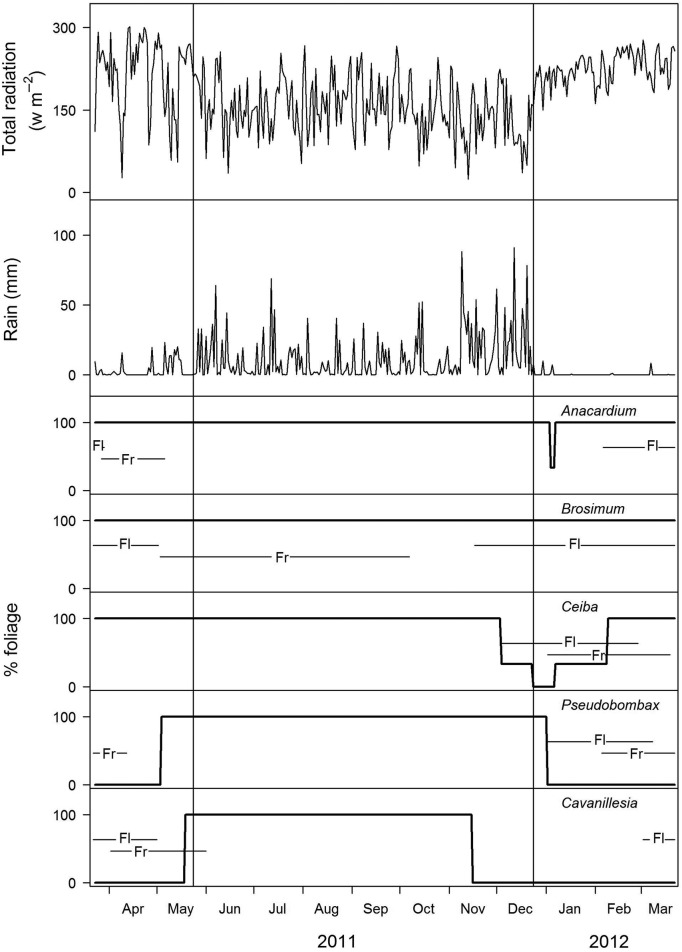
Seasonal changes in climate during the study period and long-term average phenology of the trees studied. The upper plots show daily mean radiation and daily precipitation data measured at 48 m, above the forest canopy. Thin vertical lines indicate the limits of dry and wet seasons—typically, the dry season ends earlier than in 2011. In the five tree plots: thick lines indicate leaf status, ‘Fl’ time of flowering and ‘Fr’ fruiting. Leaves of *A. excelsum* are exchanged within days, whereas *B. alicastrum* is never leafless. *Ceiba pentandra* may lose its leaves for about three months, which happens only every 4 to 5 years. *Pseudobombax septenatum* and *C. platanifolia* are drought-deciduous trees. Climate data are from a walk-up tower of the Physical Monitoring Program of STRI. Phenology data are from the Panama species database provided by the STRI website and Condit *et al.* (2011).

Five tree species were selected for the study. Selection criteria were emergent crowns, thus deciduousness would have a strong effect on microclimate; more or less even distribution across the island; accessibility with the single rope technique. The following species were selected: *Anacardium excelsum* (Bertero ex Kunth) Skeels (Anacardiaceae) and *Brosimum alicastrum* Sw. (Moraceae; both evergreen), *Ceiba pentandra* (L.) Gaertn. (Malvaceae) as a semi-deciduous species and *Pseudobombax septenatum* (Jacq.) Dugand and *Cavanillesia platanifolia* (Humb. & Bonpl.) Kunth (both deciduous Malvaceae). In the following, species are addressed by their genus names. The deciduous trees are leafless during the entire dry season (Fig. 1). Semi-deciduous *Ceiba* trees usually lose their leaves only for a few weeks, but every 4 to 5 years when flowering and fruiting the leafless phase lasts for up to 20 weeks (Windsor, unpublished). All chosen individuals (four to five per species, average height 40 m) were large canopy trees lacking lianas and were distributed over the entire island **[see Supporting Information]**. Tree crown diameter was estimated via stride length and diameter at breast height (dbh) determined with a tape measure.

All trees were accessed with the single rope technique (Perry 1978). Epiphytes on the trunk and on branches in the inner canopy could be accessed directly; those in the outer canopy were recorded with the help of binoculars from the central crown. Abundances of all epiphytes were quantified in a 90° sector of each tree crown and then extrapolated. The remaining crown was screened for additional species. Nomadic vines and hemiepiphytes (as defined by Zotz 2013) were recorded but excluded from statistical analyses unless being truly epiphytic, i.e. without contact to the ground. Species names follow Croat (1978) and Tropicos (2012). Voucher specimens were deposited in the Herbarium of the University of Panama. Since the delimitation of individual plants was often difficult, Sanford's (1968) definition of a stand was followed: a group of rhizomes, stems and leaves belonging to one species, which is clearly separated from conspecifics. Small juveniles were only included when exceeding 20 % of the maximum size of a given species.

### Microclimate

The trees we studied were canopy trees with at least partly emergent crowns. Microclimatic differences should be most pronounced within crowns, while stems are protected by both crown and surrounding vegetation leading to more homogeneous microclimatic conditions. Thus, we confined microclimate measurements to tree crowns. A crown was defined as the upper part of the tree with all major limbs, encompassing Johansson zones III–V (Johansson 1974). Total radiation in the inner crown (analogous to Johansson zone III) of each studied tree was documented for about 1 year from 22 March 2011 to 6 March 2012 with one HOBO pendant data loggers (Onset Computer Corporation, Pocasset, USA) per tree. Loggers were placed in a southerly direction within Johansson zone III. The pendant loggers measure ambient luminance in lux within a broad wavelength spectrum (ca. 150–1200 nm). Thus, the data are not numerically comparable with photosynthetic active radiation, but allow a relative comparison. As such we defined the highest measured daily mean as 100 % and expressed all other readings relative to this extreme. Readings were taken every 40 min. Loggers were calibrated against each other. For statistical analyses we calculated daily means.

Evaporation was estimated after Didham and Lawton (1999). Per tree four millilitre-scaled test tubes were filled with 10 mL of water each, a filter paper was added to enlarge the evaporating surface. The tubes were left in the crown (oriented in the four cardinal directions in Johansson zone III) for 2–6 h around noon, allowing the calculation of hourly evaporation. Because daily variation in evaporation was large and measurements were done on different days from 19 February to 24 March 2010 and from 5 March to 21 March 2011 (in *Cavanillesia*), we standardized our data. Evapotranspiration is continuously measured with an ET gauge (Spectrum Technologies, Inc., Aurora, USA) on top of a walk-up tower at a height of 48 m by the Physical Monitoring Program of STRI. In March 2011, we compared the readings obtained with our method on the tower with readings of the ET gauge on 4 days. This allowed us to standardize our data and to express evaporation measured in tree crowns as a proportion of the same day's evapotranspiration measured at the tower.

**Figure 2.**
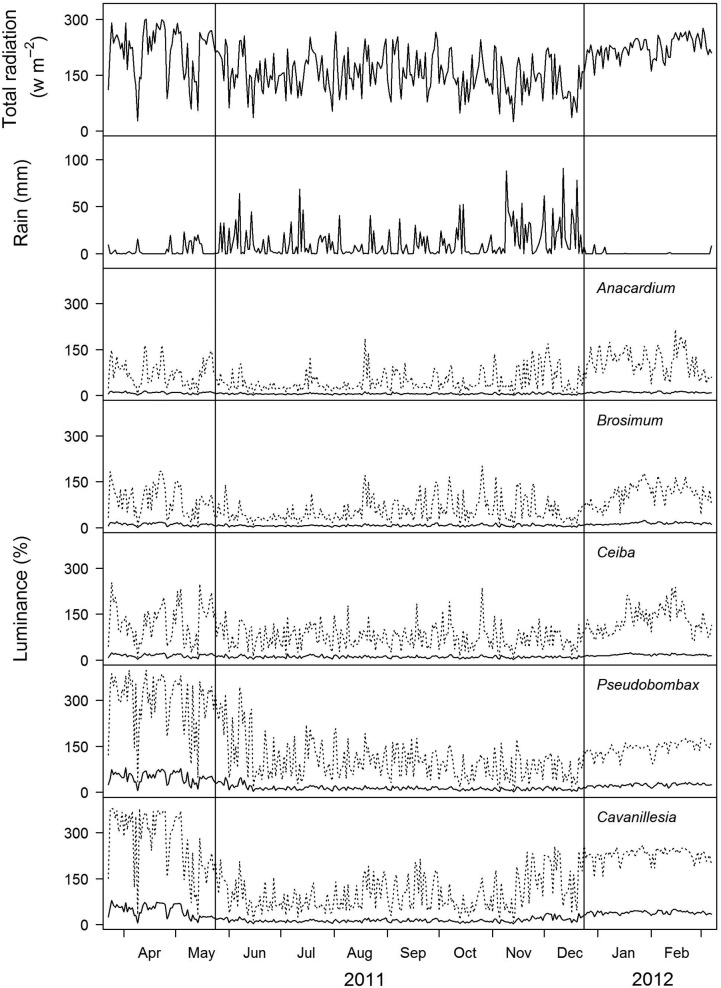
Seasonal changes in radiation and rainfall. Data for radiation and rain data were obtained from the Physical Monitoring Program of STRI. Light is presented as daily mean, while rain represents daily integrals. Daily means (solid line) of total radiation in percentage of the highest daily mean measured in the tree crowns of the five species studied (*A. excelsum*, *n* = 4 trees; *B. alicastrum*, *n* = 4; *C. pentandra*, *n* = 4; *P. septenatum*, *n* = 3; *C. platanifolia*, *n* = 4) over the course of 1 year. The dotted lines depict the mean daily maximum. The vertical lines mark the limits of dry and wet seasons (see MM for definition).

To allow comparisons of the microclimatic conditions within tree crowns, the study period was divided into dry and wet seasons. The two dry seasons covered by this study were quite distinct, but differences within the tree species were consistent. Thus, we combined the data of both dry seasons. For regions with pronounced dry periods, dry season duration is commonly defined as the period to accumulate 10 % of annual total rainfall (Reiser and Kutiel 2008). This percentage also represents the average amount reported by the Physical Monitoring Programme of STRI. The dry season on the BCI is not only characterized by lower absolute rainfall but also by a lower frequency of rainfall events. Therefore, we defined the start and the end of dry seasons according to a combination of frequency and amount of rainfall. The first dry season ended on 23 May 2011, which was the last day of a series of at least 5 days with <0.01 % of the year's total rainfall. The start of the second dry season was 24 December 2011, which was the first day of a series of at least 5 days with <0.01 % of the year's total rainfall. As expected, the total rainfall in the dry seasons was ca. 10 % of the annual total rainfall. Rainfall data from the study period are given in Fig. 2.

### Tree bark

Samples from major branches in the inner crown of four trees per species were collected by cutting off pieces of the phellem. This material was used to determine bark WHC (compare with Callaway *et al.* 2002). Briefly, samples were cleaned of any moss, oven-dried (50 °C) and their dry weight determined. Water-holding capacity was determined after soaking the samples in water for half an hour. Excess water was shaken off in a consistent manner and samples were weighed again after a 5-min pause (=maximum WHC per area). Subsequently, water loss rates were determined by keeping the samples at a constant temperature of ∼22 °C for 8 h on a lab bench and weighing them at hourly intervals and expressing the loss as percentage of the maximum WHC.

### Epiphyte growth

We selected two abundant species for growth analysis, which formed accessible populations in a reasonable subset of the study trees. Growth of individuals in populations of *Dimerandra emarginata* and *Niphidium crassifolium* was estimated between March 2011 and March 2012. These two species are generally abundant on BCI, are relatively well adapted to drought and grew in almost all surveyed trees. However, plants were not always accessible. We were able to include seven populations of *Dimerandra* in one *Brosimum*, three *Ceiba* and three *Pseudobombax* trees and eight populations of *Niphidium* in two *Brosimum*, three *Ceiba*, two *Pseudobombax* and one *Cavanillesia* trees. Populations from conspecific tree individuals were pooled for the analyses.

Initial size, i.e. the length of the longest shoot of a plant (*Dimerandra*) and length of the longest leaf (*Niphidium*), was measured; plants were marked and mapped. The original size was defined as 100 %. The following year the difference in the length of the longest shoot/leaf to first year's length was calculated as the relative positive or negative growth in per cent and analysed with initial size as a covariate. Species differences in survival and survival of the same species on different trees were analysed with Fisher's exact test for count data.

### Epiphyte physiology

A total of 509 leaf samples of 59 species were analysed (usually six replicates) for foliar nitrogen (N) concentration and δ^13^C- and δ^15^N values. δ^13^C values were used (i) to distinguish CAM- and C_3_-species, using the generally accepted value of −20 ‰ as the cut-off between these photosynthetic pathways (Zotz 2004) and (ii) among the C_3_ plants they were used as a proxy for long-term water-use efficiency in intraspecific comparisons (Van de Water *et al.* 2002) (see the next paragraph).

A large proportion (271 samples) represented five abundant species that were used to analyse intraspecific variation in SLA, δ^13^C value, δ^15^N value and leaf N concentration in relation to host tree species and microclimate. For SLA the leaf surface was estimated via pixel counting of photographs using an image processing program (ImageJ 1.45, Rasband 1997–2012) and subsequent determination of leaf dry weight. Physiological parameters in epiphytes tend to vary substantially with plant size (Zotz *et al.* 2001). Hence, we recorded the plant size and used it as a covariate in our analyses. For the orchids their stem length, and for the two ferns the length of their longest leaf were taken as a proxy for size. All five abundant epiphyte species were C_3_ plants (Zotz and Ziegler 1997): *D. emarginata* (G. Mey) Hoehne, *Maxillaria uncata* Lindl., *Scaphyglottis behrii* (Rchb. f.) Benth.& Hook. f. ex Hemsl. (all three Orchidaceae), *N. crassifolium* (L.) Lellinger (Polypodiaceae) and *Vittaria lineata* (L.) J. Sm. (Vittariaceae). These five species were selected for their high abundance that allowed us to sample them on most tree species. *Dimerandra* and *Niphidium* grew on all five study tree species and *Niphidium* could be sampled on at least one of each. *Dimerandra* could not be sampled on *Anacardium. Vittaria* grew in all but the *Cavanillesia* trees, but could not be sampled on *Pseudobombax. Maxillaria* and *Scaphyglottis* were not found on the two deciduous tree species.

### Data analysis

Statistical tests were conducted with R 2.15.2 (R Development Core Team 2012). All data was related to tree species as factor variables: microclimate, bark maximum WHC and water loss rates, epiphyte diversity (Shannon Index), percentage of CAM species within tree crowns and the foliar traits SLA, δ^13^C value, δ^15^N value and N concentration. These data were tested for normal distribution with ‘Lillifors (Kolmogorov-Smirnov) test for normality’, and homogeneity of variances was tested with the ‘Fligner-Killeen Test of Homogeneity of Variances’. When prerequisites were fulfilled we used parametric tests ANOVA and ANCOVA. Otherwise, we used the Kruskal–Wallis rank sum test (KW). Post-hoc tests were Tukey HSD for parametric data sets and Nemenyi-Damico-Wolfe-Dunn test for non-parametric data sets. Count data of survival from the growth study were analysed with Fisher's exact test for count data. The Shannon diversity index of abundance data was generated with EstimateS 8.20 (Colwell 2009). To assess the compositional similarity of epiphyte assemblages within the tree crowns (intra-specific and all species pooled) we calculated the multiple-assemblage similarity profile following Jost *et al.* (2011). This profile illustrates how differentiation of the assemblages varies between all species (*q* = 0), the typical species (*q* = 1) and the dominant species (*q* = 2). For the canonical correspondence analyses, we used Canoco 4.5 (ter Braak and Šmilauer 1997). This multivariate technique detects variations in the species assemblage caused by environmental variables. Tree species identity was incorporated in the analysis as supplementary nominal variable. Other environmental variables tested were evaporation, luminance (separated for the dry and wet seasons) and dbh as a measure of tree size. The statistical significance of the environmental variables was tested using the Monte Carlo permutation test with manual forward selection (Monte Carlo test run 9999 times). The analyses were run with abundance data and binary data (presence/absence). Scaling of data was symmetric between inter-species and inter-sample distances. The scaling type was a biplot scaling and data were log-transformed and rare species were down-weighted.

In all box plots the median is depicted as bold black bar. The box represents the inner quartile range (IQR), while whiskers extend to extreme values within the first quartile −1.5 × IQR and, respectively, within the third quartile +1.5 × IQR. Empty circles indicate values beyond this range.

## Results

### Abiotic and biotic conditions

Microclimate: Luminance was three to four times higher in deciduous vs. evergreen tree crowns in the dry season (deciduous: 36 and 43 % of highest daily mean, evergreen: 10 and 13 %; KW: *P* < 0.001, *χ*² = 1228.9, df = 4; Table 1, Fig. 2). The lowest luminance was observed in *Anacardium* and the highest in *Cavanillesia* (Table 1). These differences between deciduous and evergreen trees persisted during the wet season but were less pronounced. Luminance in the inner crowns of drought-deciduous trees with full foliage exceeded that in evergreen trees by about 2-fold (deciduous: 14 % of highest daily mean, evergreen: 6 and 7 %; KW: *P* < 0.001, *χ*² = 1418.7, df = 4, Table 1).

**Table 1.** Mean luminance and evaporation in the study trees during dry and wet seasons. Superscript lower case letters indicate significant differences between trees (luminance KW: *P* < 0.001, *n* per species = 785–1404; evaporation ANOVA: *P* < 0.05, *F*_(4,17)_ = 4.0). Luminance was calculated as percentage of the highest daily mean. Evaporation was calculated in relation to measurements with an ET gauge of STRI (see MM for details). ^+^ Evergreen, ° semi-deciduous, ^−^ deciduous species.

Species	Dry season		Wet season
	Luminance (%)	Evaporation (%)	Luminance (%)
*A. excelsum*^+^	9.7^a^	58^a^	5.5^a^
*B. alicastrum*^+^	13.1^b^	76^ab^	7.1^b^
*C. pentandra*°	16.2^c^	69^ab^	10.1^c^
*P. septenatum*^−^	35.9^d^	101^ab^	13.5^d^
*C. platanifolia*^−^	42.5^e^	121^b^	13.8^d^

Average evaporation, measured only in the dry season, was about twice as high in deciduous trees compared with evergreen ones, but means were only significantly different between *Cavanillesia* and *Anacardium* trees (ANOVA: *P* < 0.05, *F*_(4,17)_ = 4.0; Table 1).

Bark: Species differences in water storage capacity of the bark were unrelated to leaf phenology. Maximum water content ranged from 20 to 144 mg cm^−^². Each pair of evergreen and deciduous trees had one species with relatively high and one with relatively low WHC (Table 2). However, these trends were not significant (KW: *P* = 0.08, *χ*² = 8.3, df = 4). On the other hand, rates of water loss tended to be highest in the deciduous species. Water loss after 8 h was significantly higher for *Ceiba* and *Cavanillesia* compared with *Brosimum* (ANOVA: *P* < 0.05, *F*_(4,15)_ = 4.8; Table 2).

**Table 2.** Water-holding capacity and water loss of the bark of five tree species. Values are means ± SD (*n* = 4) for each tree species (*A. excelsum* (Ana), *B. alicastrum* (Bro), *C. pentandra* (Cei), *P. septenatum* (Pse), *C. platanifolia* (Cav)). Significant differences are indicated by superscript lower case letters (ANOVA: *P* < 0.05, Tukey HSD).

	Ana	Bro	Cei	Pse	Cav
Bark WHC (mL cm^−2^)	94.0 ± 37.7	36.0 ± 15.5	64.5 ± 22.1	90.3 ± 61.7	33.3 ± 7.5
Bark water loss (%)	62.0 ± 6.7^ab^	52.8 ± 13.6^a^	74.3 ± 5.2^b^	65.8 ± 7.8^ab^	75.8 ± 7.3^b^

### Epiphyte flora and assemblages

In total, the 24 studied trees were host to an estimated 26 000 individual epiphytes ( = stands) from 83 holoepiphyte species, two hemiepiphyte species and two nomadic vines, which had no contact with the ground **[see Supporting Information** for a complete list**]**. These represented 16 families (Table 3). Three families accounted for more than two-thirds of all species, i.e. Orchidaceae (40 %, 35 spp.), Araceae (13 %, 11 spp.) and Bromeliaceae (10 %, 9 spp.). Ranking families by abundance leads to relatively small changes. Orchidaceae was even more prominent (56 %), followed by four families with at least 5 % of all individuals: Polypodiaceae (11 %), Dryopteridaceae, Araceae (both 8 %) and Bromeliaceae (5 %).

**Table 3.** Epiphyte abundance and diversity in the trees studied. Shown are families of epiphytes found in the five tree species studied: *A. excelsum* (Ana, *n* = 5), *B. alicastrum* (Bro, *n* = 4), *C. pentandra* (Cei, *n* = 5), *P. septenatum* (Pse, *n* = 5) and *C. platanifolia* (Cav, *n* = 5). Species numbers are given in parentheses. Prevalence of CAM was calculated for each column's total excluding nomadic vines and hemiepiphytes. Data summarize epiphytes on a given tree, except ‘CAM species—crown’ and ‘Shannon index’ that refer to crown data only. Significant differences are indicated by superscript lower case letters (ANOVA: *P* < 0.05, Tukey HSD). *Including two species of nomadic vines. ^†^Hemiepiphyte.

	Individuals (species)	Ana	Bro	Cei	Pse	Cav
Total	25 592 (87)	10 963 (65)	3432 (49)	8826 (50)	2296 (20)	75 (7)
Araceae*	2050 (11)	1504 (10)	288 (8)	249 (7)	7 (4)	2 (2)
Aspleniaceae	564 (2)	554 (2)	5 (1)	5 (1)	0 (0)	0 (0)
Bromeliaceae	1398 (9)	1041 (7)	201 (2)	154 (3)	2 (2)	0 (0)
Cactaceae	84 (2)	5 (1)	53 (2)	26 (2)	0 (0)	0 (0)
Clusiaceae^†^	118 (1)	19 (1)	23 (1)	76 (1)	0 (0)	0 (0)
Cyclanthaceae	218 (1)	8 (1)	0 (0)	210 (1)	0 (0)	0 (0)
Davalliaceae	379 (1)	139 (1)	23 (1)	217 (1)	0 (0)	0 (0)
Dryopteridaceae	2135 (6)	1827 (6)	2 (1)	305 (2)	1 (1)	0 (0)
Gesneriaceae	377 (1)	0 (0)	377 (1)	0 (0)	0 (0)	0 (0)
Hymenophyllaceae	30 (2)	30 (2)	0 (0)	0 (0)	0 (0)	0 (0)
Lycopodiaceae	14 (1)	5 (1)	5 (1)	4 (1)	0 (0)	0 (0)
Orchidaceae	14 329 (35)	5128 (23)	1701 (19)	5486 (21)	1982 (10)	32 (2)
Piperaceae	630 (5)	177 (2)	87 (4)	363 (2)	0 (0)	3 (1)
Polypodiaceae	2778 (7)	325 (5)	560 (6)	1564 (5)	291 (2)	38 (2)
Rubiaceae^†^	132 (1)	99 (1)	5 (1)	28 (1)	0 (0)	0 (0)
Vittariaceae	356 (2)	102 (2)	102 (1)	139 (2)	13 (1)	0 (0)
CAM plants	1242 (14)	117 (6)	319 (11)	401 (10)	374 (3)	31 (1)
CAM individuals (%)	4.9	1.1	9.3	4.5	16.3	41.3
CAM species (%)	16.0	9.0	21.2	18.5	13.6	14.3
CAM species—crown (%)		6.6 ± 5.5^a^	18.5 ± 15.7^ab^	22.6 ± 12.0^ab^	31.3 ± 6.4^b^	8.3 ± 16.7^ab^
Shannon index		2.1 ± 0.6^a^	2.0 ± 0.7^a^	1.9 ± 0.7^a^	0.7 ± 0.3 ^b^	0.4 ± 0.5^b^

Three epiphyte species have not been documented previously for BCI (*Elaphoglossum* cf. *doanense, E. peltatum* and *E. latum*—all Dryopteridaceae), while the fourth, *Maxillaria acervata* (Orchidaceae), had been collected twice by the last author in recent years, without publishing this finding.

The largest number of species was found on the five *Anacardium* trees (65 species), which represents more than two-thirds of all epiphyte species found in this study. In *Brosimum* and *Ceiba* trees, we still found 49 and 50 species, respectively. The deciduous *Pseudobombax* and *Cavanillesia* supported considerably fewer epiphytes with only 20 and 7 species, respectively. None of the epiphyte species found on the deciduous trees were restricted to these tree species.

The results shown so far represent the assemblages of the entire trees. Epiphytes growing on the trunks of emergent trees are less affected by tree phenology since they are protected by the crown and the surrounding vegetation. Therefore, we expected differences in the epiphyte assemblages to be most pronounced or even to be confined to the emergent crowns. Indeed we did not find significant differences in epiphyte diversity on the trunk between tree species (ANOVA: *P* = 0.5, *F*_(4,14)_ = 0.9), and excluded all epiphytes growing on the main trunks from further analysis. The average epiphyte diversity in the crowns of the evergreen and semi-deciduous species was ∼4-fold higher compared with the two deciduous tree species (ANOVA: *P* < 0.001, *F*_(4,19)_ = 9.8; Table 3). Likewise, differences in epiphyte abundance among tree crowns were also significant (KW: *P* < 0.01, *χ*² = 13.6, df = 4; data not shown). Deciduous trees hosted fewer individuals than evergreen and semi-deciduous trees, although the difference was only significant between deciduous *Cavanillesia* and both evergreen *Anacardium* and semi-deciduous *Ceiba*.

**Figure 3.**
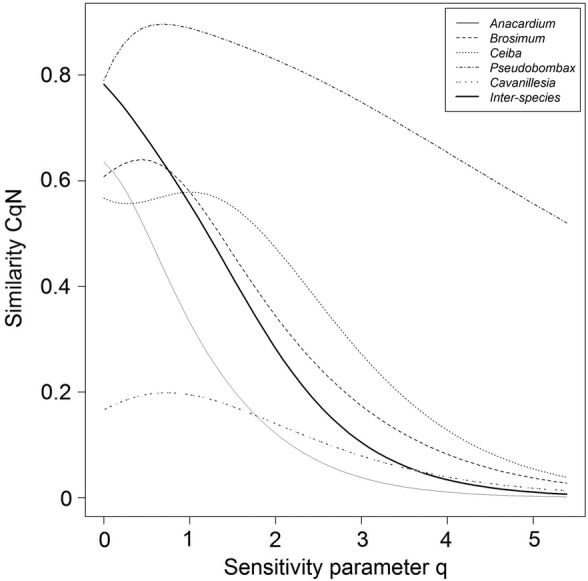
The multiple-assemblage similarity profile CqN (for *q* = 0–5) of epiphyte assemblages in the tree crowns of *A. excelsum* (5 trees/60 epiphyte species), *B. alicastrum* (4/43), *C. pentandra* (5/47), *P. septenatum* (5/7), *C. platanifolia* (4/7) and of epiphyte assemblages in tree crowns between all trees (23/79).

The consistency of the most abundant species between individual tree crowns of a species is indicated by the sensitivity parameter *q* = 2. *Anacardium* showed the least congruence (12 %) while most abundant species' identity was highly consistent in *Pseudobombax* (83 %, Fig. 3). Congruence of abundant species increased from *Brosimum* (34 %) to *Ceiba* (47 %). Deciduous *Cavanillesia* showed very low congruence with 14 % unlike its counterpart *Pseudobombax*. However, they were similar in respect to the high evenness in the species occurrence among their tree crowns, which is reflected in the less steep slopes of their similarity profile compared with the evergreen and semideciduous trees. All tree individuals pooled shared ∼28 % of the most abundant species in their crowns.

**Figure 4.**
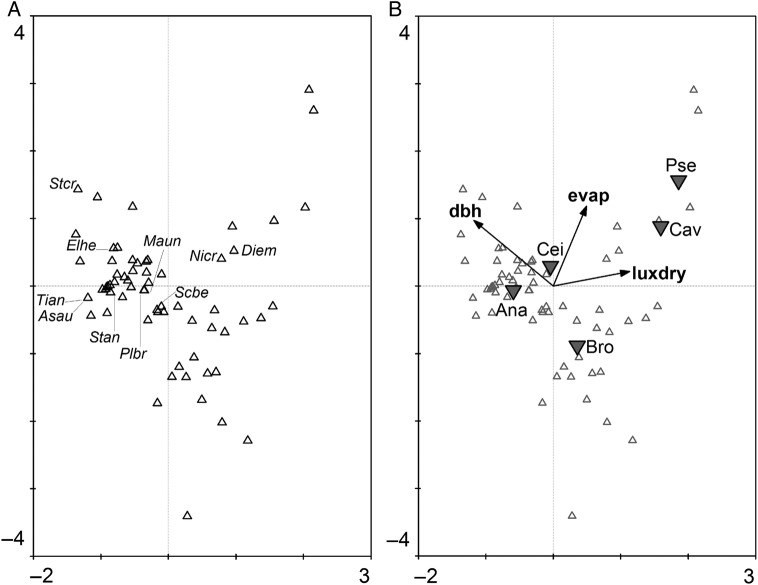
Species-conditional triplot (divided into two graphs for easy interpretation) based on a canonical correspondence analysis of the log-transformed epiphyte species abundance data (rare species were down-weighted) displaying 18 % of the inertia (=weighted variance) in the abundances and 84 % of variance in the weighted averages and class totals of species with respect to environmental variables. The eigenvalues of Axis 1 (horizontally) and Axis 2 (vertically) are 0.26 and 0.15, respectively. Displayed are species (small triangles) and tree centroids (down triangles). Quantitative environmental variables are indicated by arrows in (B) (dbh of trees, evaporation and luminance measured in dry season). In the analysis 79 true epiphyte species were included; only the 10 most abundant species are labelled in (A). Epiphyte species names are abbreviated with the first two letters of the genus and epitheton: *Asplenium auritum*, *Dimerandra emarginata*, *Elaphoglossum herminieri*, *Maxillaria uncata*, *Niphidium crassifolium*, *Pleurothallis brighamii*, *Scaphyglottis behrii*, *Stenospermation angustifolium*, *Stelis crescentiicola* and *Tillandsia anceps*. Tree species are: *A. excelsum*, *B. alicastrum*, *C. pentandra*, *P. septenatum* and *C. platanifolia*.

The differentiation of epiphyte assemblages in evergreen and deciduous tree crowns was also reflected by a canonical correspondence analysis (Fig. 4). When environmental variables were added, luminance during the dry season and evaporation contributed significantly to the explanation of the variation in species data. Diameter at breast height as a proxy for tree size/age was also significant. An analysis with binary data yielded very similar results although evaporation had no significant influence anymore (data not shown).

### Crassulacean acid metabolism

We compared the percentages of CAM-performing species and individuals in different tree crown assemblages to address functional changes apart from floristic ones. Disregarding *Cavanillesia*, there was a gradual increase in CAM species from evergreen, semi-deciduous to deciduous tree crowns. The difference in the proportion of CAM species between deciduous *Pseudobombax* and evergreen *Anacardium* was significant (ANOVA: *P* < 0.05, *F*_(4,18)_ = 3.7; Table 3). The low percentage of CAM epiphytes in *Cavanillesia* crowns (33 % in one tree) is probably a sampling artefact, since *Cavanillesia* hosts very few individuals and species in general: we never found more than eight individuals from three species in any tree (Table 3). Xerophytic C_3_ species such as *N. crassifolium* or *Polypodium polypodioides* were the only epiphytes in three of five *Cavanillesia* crowns. There was no significant difference in the abundance of CAM epiphytes with tree species although the percentage of CAM-performing individuals could reach >50 % in deciduous tree crowns, while evergreen and semi-deciduous trees never hosted >20 % in their crowns (data not shown).

### Individual performance and microclimatic differences

Growth and survival were studied in *Dimerandra* and *Niphidium*. Survival was significantly lower for *Dimerandra* than for *Niphidium* (Fisher's exact test: *P* < 0.05). Tree identity did not affect the survival or growth of *Dimerandra* (Fisher's exact test: *P* = 0.6; KW: *P* = 0.08, *χ*² = 5.1, df = 2) but survival and growth of *Niphidium* differed significantly among trees (Fisher's exact test: *P* < 0.05; ANOVA: *P* < 0.001, *F*_(3,40)_ = 12.6; **[see Supporting Information]**).

*Dimerandra*'s mean annual relative growth rate (RGR) ranged from 8 % in *Ceiba* to 42 % in *Pseudobombax*. This coincides with the prevalence of *Dimerandra* in *Pseudobombax* crowns where it accounted for 70 % of all epiphytes. Survival of *Niphidium* was much lower in *Pseudobombax* (only 5 of 13 individuals were still alive in 2012) compared with that in both *Brosimum* (24 of 25 individuals survived) and *Ceiba* (22 of 26 individuals survived). Also indicative of harsh conditions in deciduous *Pseudobombax* were the slow RGRs of *Niphidium*.

### Intraspecific differences in SLA, *δ*^13^C, *δ*^15^N and N concentration of leaves in relation to microclimatic differences

Possible differences among populations of the same species associated with microclimatic differences were assessed for several leaf traits (SLA, δ^13^C, δ^15^N and leaf N concentration) in *D. emarginata*, *Maxillaria uncata*, *N. crassifolium*, *Scaphyglottis behrii* and *V. lineata*.

We used size as a covariate in our analyses as it has been shown to influence physiological parameters of epiphytes (Zotz *et al*. 2001). Our results, however, were very inconsistent between the various species regarding whether the influence was significant for a given parameter and occasionally also regarding the direction of the correlation when a significant influence was found **[see Supporting Information]**.

**Figure 5.**
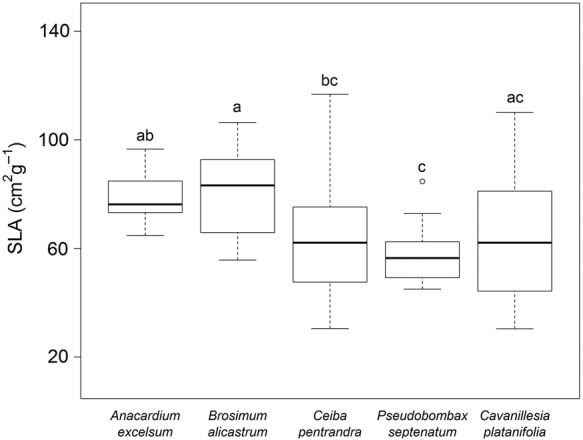
Specific leaf area of *N. crassifolium* individuals (83) sampled on different tree species. Letters indicate significant differences (ANCOVA with plant size as the covariate: *P* < 0.05, *F*_(4,73)_ = 5.8).

There was a trend towards lower SLA in deciduous trees for *Niphidium* (ANCOVA: *P* < 0.001, *F*_(4,73)_ = 5.8, Fig. 5). Specific leaf area of *Dimerandra* and *Vittaria* also showed significant differences between tree species but without a clear pattern and the result was blurred by a significant interaction with size.

**Figure 6.**
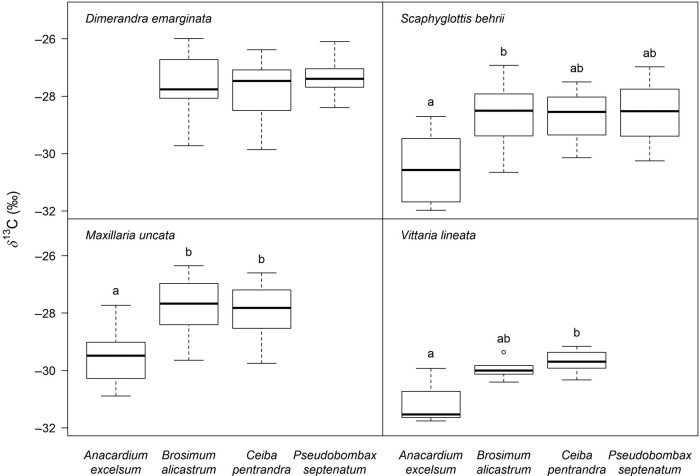
δ^13^C values for *Dimerandra* (66), *Maxillaria* (40), *Scaphyglottis* (27) and *Vittaria* (15) sampled on different tree species. Letters indicate significant differences (ANCOVA with plant size as the covariate: *Maxillaria P* < 0.05, *F*_(1,36)_ = 6.2 and *Scaphyglottis P* < 0.05, *F*_(3,22)_ = 5.4; ANOVA: *Vittaria P* < 0.05, *F*_(2,11)_ = 5.5).

In deciduous trees, δ^13^C was generally less negative in *Scaphyglottis* (Fig. 6). *Niphidium* also showed the same trend, but interpretation is difficult because of a significant interaction with relative plant size. *Maxillaria* and *Vittaria* were not found in deciduous trees, while *Dimerandra* showed no significant differences in δ^13^C values between the trees included.

**Figure 7.**
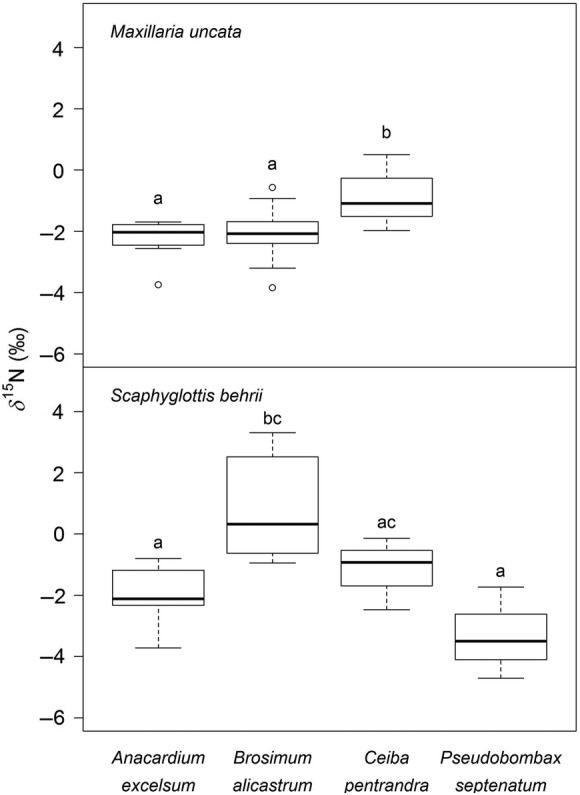
δ^15^N values for *Maxillaria* (38) and *Scaphyglottis* (28) sampled in different tree crowns. Letters indicate significant differences (ANCOVA with plant size as the covariate: *Maxillaria P* < 0.05, *F*_(1,33)_ = 15.3; ANOVA: *Scaphyglottis P* < 0.05, *F*_(3,23)_ = 13.9).

The only species with significant differences in δ^15^N values between trees was *Scaphyglottis*, which showed a tendency towards more negative values when growing in deciduous tree crowns (Fig. 7). *Dimerandra* and *Niphidium* showed consistent results, with higher δ^15^N values in evergreen *Brosimum* than in semi-deciduous *Ceiba* and deciduous *Pseudobombax*. δ^15^N values for *Niphidium* in *Anacardium* and *Cavanillesia* did not vary from those of plants growing in *Ceiba*, however. We were not able to obtain samples of *Dimerandra* from *Anacardium* or *Cavanillesia*. Yet there was a significant interaction of host tree species with relative plant size of *Dimerandra* and *Niphidium. Maxillaria* was not found in deciduous trees.

The only species with significant differences in leaf N concentrations between trees were *Niphidium* (ANOVA: *P* < 0.001, *F*_(4,74)_ = 8.1) and *Dimerandra*. But for the latter there was a significant interaction with relative size and for *Niphidium* there was no clear tendency or grouping with leaf phenology **[see Supporting Information]**. No size-related variation was found.

## Discussion

This study is based on the understanding that the composition of a community is strongly influenced by abiotic and biotic factors and tries to identify general rules to explain distributional patterns of vascular epiphytes in time and space (Díaz *et al.* 1999). This approach contrasts with the neutral theory (Hubbell 2001), which emphasizes the importance of stochastic processes. From a deterministic point of view the environment acts as a filter that governs the final community composition at a given location (Keddy 1992). Zobel's (1997) distinction of three different species pools (actual, local and regional) provides a useful conceptual framework for the comparison of epiphyte assemblages found on each of our five study species (=actual species pool) vs. those reported for BCI (=regional species pool). The 92 species (including five nomadic vines that had ground contact) occurring on the 24 studied trees represent almost half of the entire epiphyte flora of BCI (15.6 km^2^, Croat 1978). This large number is comparable with the number of species found on 12 similarly large trees at the San Lorenzo Canopy Crane site (83 species, Zotz and Bader 2011) located ∼20 km as the crow flies from BCI in a much wetter climatic setting. Additionally, we found three ferns and an orchid species, which are not included in the flora of BCI by Croat (1978). Although we found a large number of species on the few study trees, more than 100 epiphyte species of the regional species pool were not covered by our study. Some of these ‘missing’ epiphyte species would probably be found with more extensive sampling on other conspecific trees, but others may indeed be excluded by particular traits of the studied tree species. Leaf phenological patterns can arguably act as a potent filter for the local species pool. We analysed its influence at different levels. In the composition of epiphyte assemblages, we found more drought-adapted species in deciduous trees. At the level of physiological plasticity some species showed adjustment to drier conditions and reduced growth performance in populations of individual species growing in deciduous trees. Besides documenting these effects, we also identified the mechanism: luminance and evaporation showed the influence of host tree leaf phenology on the microclimate in tree crowns, while bark characteristics seem unrelated.

As expected, we found marked differences in microclimatic conditions during the dry season between evergreen and deciduous trees, e.g. much higher luminance and much higher evaporative demand in deciduous tree crowns. The number of epiphyte species, which thrive under such demanding conditions, is obviously limited. Thus, both species diversity and epiphyte abundance were significantly reduced in deciduous trees. A multiple-assemblage similarity profile (Fig. 3) indicated that epiphyte assemblages of the deciduous trees (*Pseudobombax*) change less in respect to their most dominant species, and epiphyte abundances were also more even. In contrast, epiphyte assemblages of evergreen trees shared fewer abundant species, and had a lower percentage of abundant species. This is typical for species-rich communities with few abundant but many rare species. Averaged over all tree individuals trees shared more abundant species than did *Anacardium* individuals at an intra-specific level.

In agreement with our results, the percent cover of vascular epiphytes in an evergreen tree species (*Hyeronima alchorneoides*) was significantly higher compared with a deciduous tree species in La Selva, Costa Rica (*Lecythis ampla*, Cardelus 2007). Both studies consistently indicate that epiphytes growing in deciduous trees are also generally found in evergreen trees (Cardelus 2007). An effect of deciduousness on epiphyte assemblages in the wet forest at La Selva is somewhat surprising, because there precipitation exceeds 100 mm per month even during the short dry season (Cardelus and Chazdon 2005). Moreover, the studied tree species were deciduous during the rainy season. This suggests that the persistent differences between deciduous and evergreen trees in luminance, and possibly other microclimatic variables in the rainy season on BCI are relevant as well.

Tree phenology did not only affect the floristic composition of epiphyte assemblages (Figs 3 and 4). At a functional level, there was a general increase in the proportion of species using the water-conserving CAM-pathway, from evergreen *Anacardium* over semi-evergreen *Ceiba* to drought-deciduous *Pseudobombax* (Table 3). The seeming deviation of *Cavanillesia* is probably a sampling artefact due to the low number of species found in the crowns. At the level of the entire *Cavanillesia* trees, >40 % of all individuals used CAM, compared with an average of ca. 5 % in the two evergreen species (Table 3). In summary, the drought-deciduous tree species offer drier growth conditions for epiphytes throughout the year, which leads to impoverished epiphyte assemblages with a large proportion of CAM species.

The studied components of microclimate are clearly not the sole factors affecting the diversity of these epiphyte assemblages, e.g. *Pseudobombax* supported more diverse assemblages compared with *Cavanillesia*. Bark water storage capacity is generally assumed to be important for epiphytes and, Barkman (1958) listed it as a key trait apart from bark pH, bark electrolyte concentration and bark buffer capacity. The highest maximum water content we measured exceeded that reported by Callaway *et al.* (2002) by more than a third, but maximum WHC of tree bark did not correlate with either epiphyte diversity or abundance. In another study communities of epiphytic bryophytes could be related to some extent to the water storage capacity of the host (Studlar 1982). However, water storage capacity greatly varies even within single tree species as the bark structure varies with age and position in the tree, and our data thus have to be interpreted with caution. Water loss rates of the bark samples in our study, which were within the range of the values reported by Callaway *et al.* (2002), may have more explanatory power: semi-deciduous and deciduous trees had consistently higher water loss rates compared with evergreen trees, which should reinforce any effect associated with phenology. We have recently shown that small differences in water supply may be crucial during germination under the demanding conditions of tree canopies (Wester and Zotz 2011). Contrary to Callaway *et al.* (2002) and Studlar (1982), we did not find an association of water storage capacity of host tree bark and epiphyte diversity. As there was no consistent correlation of the water storage of tree bark and phenology, this aspect did not enhance the phenology effect whereas differences in water loss rates arguably did. We conclude that water storage capacity is a minor filter in our system.

We also expected differences in functional attributes of conspecifics growing on trees with varying phenology. ^13^C in tissues can be a good proxy for whole plant water-use efficiency (Cernusak *et al.* 2008), more positive δ^13^C values being generally associated with drier microclimates (e.g. Lajtha and Getz 1993; Van de Water *et al.* 2002). Leaf structure, i.e. SLA, is also known to respond to growing conditions (Cornelissen *et al.* 2003). Expectations only partly bore out. A slight trend towards lower SLA was found in only one of five species, but δ^13^C values of most species were indeed more positive in deciduous tree crowns. Thus, although not entirely consistent, there are detectable intraspecific differences in functional traits of epiphyte populations in trees with different leaf phenology.

Plant size has been identified as an important covariate in physiological studies with epiphytes (Schmidt *et al.* 2001). Since an increase in plant size almost inevitably leads to a decrease in surface/volume ratios, plant water relations must be affected, e.g. because identical transpiration rates would deplete stored water more rapidly in smaller plants under otherwise identical conditions (Schmidt *et al.* 2001). This would immediately result in a less favourable water status in smaller plants. Possible adaptive responses are reduced SLA or reduced stomatal conductance in small plants. However, we found no consistent size dependence, neither for SLA nor for δ^13^C, other local factors obviously being of overriding importance.

The mean δ^15^N value of all tested epiphytes of our study was in the range of a study including epiphytes from Brazil, Australia and Solomon Islands (Stewart *et al.* 1995) and slightly lower δ^15^N values were reported for Costa Rican and Mexican epiphytes (Hietz *et al.* 2002; Hietz and Wanek 2003). Epiphytes are supplied with N from various sources, e.g. via dry and wet deposition of reactive N from the atmosphere, biological N_2_ fixation and decomposition of dead canopy organic material (Benzing 1989). Since different sources can differ in their δ^15^N signatures (Robinson 2001), the δ^15^N values of epiphyte tissue can be used to infer their main N source. Atmospheric N has, by definition, a δ^15^N value of 0‰ and biologically fixed N therefore commonly has isotope signatures close to this. The isotopic signatures of N inputs from dry and wet deposition are highly variable (e.g. Lindberg *et al.* 1986; Heaton *et al.* 1997; Högberg, 1997) but δ^15^N values tend to be higher in dry than in wet deposition (Moore 1977; Garten 1992; Heaton *et al.* 1997). Low δ^15^N signatures in epiphytes have therefore been explained by efficient use of atmospheric wet deposition (Stewart *et al.* 1995). Tree foliage captures dry deposition of reactive N forms and channels it into the inner crown to the epiphytes during rain events. This scenario should result in a larger yearly portion of dry deposition in evergreen trees than in deciduous trees. Consequently, we expected epiphytes in evergreen trees to show less negative δ^15^N values compared with deciduous trees. Less atmospheric N deposition could also result in lower leaf N concentrations of epiphytes in deciduous trees. However, any such effect could easily be confounded by reduced growth due to drought stress. Of the five studied epiphyte species only one showed the expected trend, which can hardly be taken as strong support for the scenario outlined above. Regarding N, our results showed no consistent pattern either. This might partly be due to drought-induced growth inhibition, which we actually documented for one species in *Pseudobombax*. Overall, our data do not allow us to make strong conclusions on possible effects of leaf phenology on the N cycle in tree canopies.

Individual growth performance integrates all physiological processes that change in response to the environment and thus integrates the environmental influence. This has been e.g. shown in a 7-year study of the population dynamics of the epiphytic orchid *Aspasia principissa* (Zotz and Schmidt 2006). Intraspecific variation at the level of individual growth was studied in two species. Differences in growth were inconsistent. While *Niphidium* was hampered in its growth in deciduous trees, the opposite trend was observed for *Dimerandra*. Species that can cope with the drier conditions in deciduous trees can take advantage of increased radiation. *Dimerandra* is a good example for this. This C_3_ species copes well with drier microclimate and obviously prefers more exposed sites. Although it does grow in evergreen trees, it almost always grew in the outermost sections (Johansson zone V) of *Anacardium* crowns. Tolerance to severe drought of this light-demanding species also explains its abundance in many *Pseudobombax* crowns. Therefore, its overall poor survival compared with *Niphidium* is rather surprising. No differences regarding the survival or growth rate among populations were found between different tree species. In contrast, survival and growth of *Niphidium* were lowest in deciduous trees. Thus, it was possible to relate microclimatic conditions to intraspecific growth differences at least for one species. Species that dominated in the drier climate of deciduous trees were not limited by more humid climate of evergreen trees, as they did occur in evergreen trees as well. We did observe that these species grew preferentially in the drier and lighter parts of the evergreen tree crowns. Thus, the drier climate of deciduous tree crowns poses a hard filter for all species that cannot cope with these conditions. Similarly, Larrea and Werner (2010) and Toledo-Aceves *et al.* (2012) found impoverished epiphyte communities in remnant or shade trees drier compared with fragments of humid montane forest. In contrast, the more humid climate of evergreen trees poses only a partial filter to drought-tolerant epiphytes.

We included *C. pentandra* in this study because a semi-deciduous tree should offer intermediate growth conditions. Consistent with this expectation, many aspects studied in this report, from species composition to physiological traits, were intermediate between evergreen and deciduous trees. In many regards, the combination of a rather open, only briefly leafless, crown with large limbs makes *Ceiba* a relatively good host for vascular epiphytes (Valdivia 1977).

## Conclusions

To conclude, we found that tree phenology imposes an imprint on epiphyte assemblages at different levels of integration, from physiology to species composition of epiphytes on individual trees. The effect of host tree phenology was not always clear-cut and entirely consistent, but is arguably modified by many other factors, only some of which could be studied. Neither dismissing the importance of these additional filters nor dismissing the role of chance our study describes one important environmental filter that structures the local communities of vascular epiphytes.

## Sources of Funding

Financial support from the Smithsonian Tropical Research Institute for field work in Panama is acknowledged.

## Contributions by the Authors

G.Z. conceived the study design. H.J.R.E. and J.B. performed field work and data analyses. F.H. and W.W. did isotope analyses. H.E. and G.Z. wrote the paper, which was reviewed and revised by all other authors.

## Conflicts of Interest Statement

None declared.

## Supporting Information

The following Supporting Information is available in the online version of this article –

**Figure S1.** Map of Barro Colorado Island (BCI, modified after Kinner & Paton 1998) showing the approximate locations of the study trees. Evergreen trees are triangles: *Anacardium excelsum* (blue) and *Brosimum alicastrum* (green); circles represent the semi-evergreen *Ceiba pentandra*; quadrats represent the drought-deciduous species: *Pseudobombax septenatum* (yellow) and *Cavanillesia platanifolia* (purple). The large yellow rectangle represents the 50-ha plot.

**Figure S2.** Growth of *Niphidium crassifolium* in four tree species measured from 2011 to 2012 (ANOVA: *P* < 0.001, *F*_(3,40)_ = 12.6). *Brosimum* (*n* = 2 trees), *Ceiba* (*n* = 4), *Pseudobombax* (*n* = 3) and *Cavanillesia* (*n* = 1).

**Figure S3.** Leaf nitrogen (N) concentration of *N. crassifolium* (81) sampled in different tree crowns. Letters indicate significant differences (ANOVA: *P* < 0.001, *F*_(4,74)_ = 8.1).

**Table S1.** Species abundances of epiphytes and hemiepiphytes. The studied species were *A. excelsum* (*n* = 5), *B. alicastrum* (*n* = 4), *C. pentandra* (*n* = 5), *P. septenatum* (*n* = 5) and *C. platanifolia* (*n* = 5). Nutrient concentrations are usually averages of six replicates, exceptional cases with a single sample are shown in italics. Some δ^13^C values were taken from other publications (^1^ Zotz & Ziegler 1997; ² Zotz 2004). δ^13^C and δ^15^N in ‰, C and N in %. * Hemiepiphyte or nomadic vine *sensu* Zotz (2013). † Nomadic vines with established ground contact via roots (excluded from further analyses).

**Table S2.** Summary of ANCOVA results for size dependence of four leaf traits. Analysed were size and its significant interaction (*) with ‘host species’ of the studied leaf traits (SLA, δ^13^C, δ^15^N, N) for the focal epiphyte species (all studied host species, *A. excelsum*, *B. alicastrum*, *C. pentandra*, *P. septenatum*, *C. platanifolia*, other = all other except the specified). ↑ Positive correlation, ↓ negative correlation, – no correlation.

Additional Information
