# Supplementary material for: Host tree phenology affects vascular epiphytes at the physiological, demographic and community level
Source: AoB Plants. 2014 Nov 11;7:plu073. doi: 10.1093/aobpla/plu073 (PMC4287691; doi:10.1093/aobpla/plu073)
Supplement: Additional Information [file supp_7_plu073_index.html]

Host tree phenology affects vascular epiphytes at the physiological, demographic and community level — Additional Information 

# Host tree phenology affects vascular epiphytes at the physiological, demographic and community level

## Additional Information

Additional Information

**Files in this Data Supplement:**

- Additional Information - Docx file
- Supplementary Table 1 - docx file
- Supplementary Table 2 - docx file
