# Supplementary material for: Host tree phenology affects vascular epiphytes at the physiological, demographic and community level
Source: AoB Plants. 2014 Nov 11;7:plu073. doi: 10.1093/aobpla/plu073 (PMC4287691; doi:10.1093/aobpla/plu073)

**Figure S1**. Map of Barro Colorado Island (modified after Kinner & Paton 1998) showing the approximate locations of the study trees. Evergreen trees are triangles: *Anacardium excelsum* (blue) and *Brosimum alicastrum* (green); circles represent the semi-evergreen *Ceiba pentandra*; Quadrats represent the drought-deciduous species: *Pseudobombax septenatum* (yellow) and *Cavanillesia platanifolia* (purple). The large yellow rectangle represents the 50 ha plot.

**Figure S2**. Growth of *Niphidium crassifolium* in four tree species measured from 2011 to 2012 (ANOVA: p < 0.001, F_(3,40)_ = 12.6). *Brosimum* (n = 2 trees), *Ceiba* (n = 4), *Pseudobombax* (n = 3) and *Cavanillesia* (n = 1).

**Figure S3**. Leaf nitrogen concentration of *Niphidium crassifolium* (81) sampled in the different tree crowns. Letters indicate significant differences (ANOVA: p < 0.001, F_(4,74)_ = 8.1).


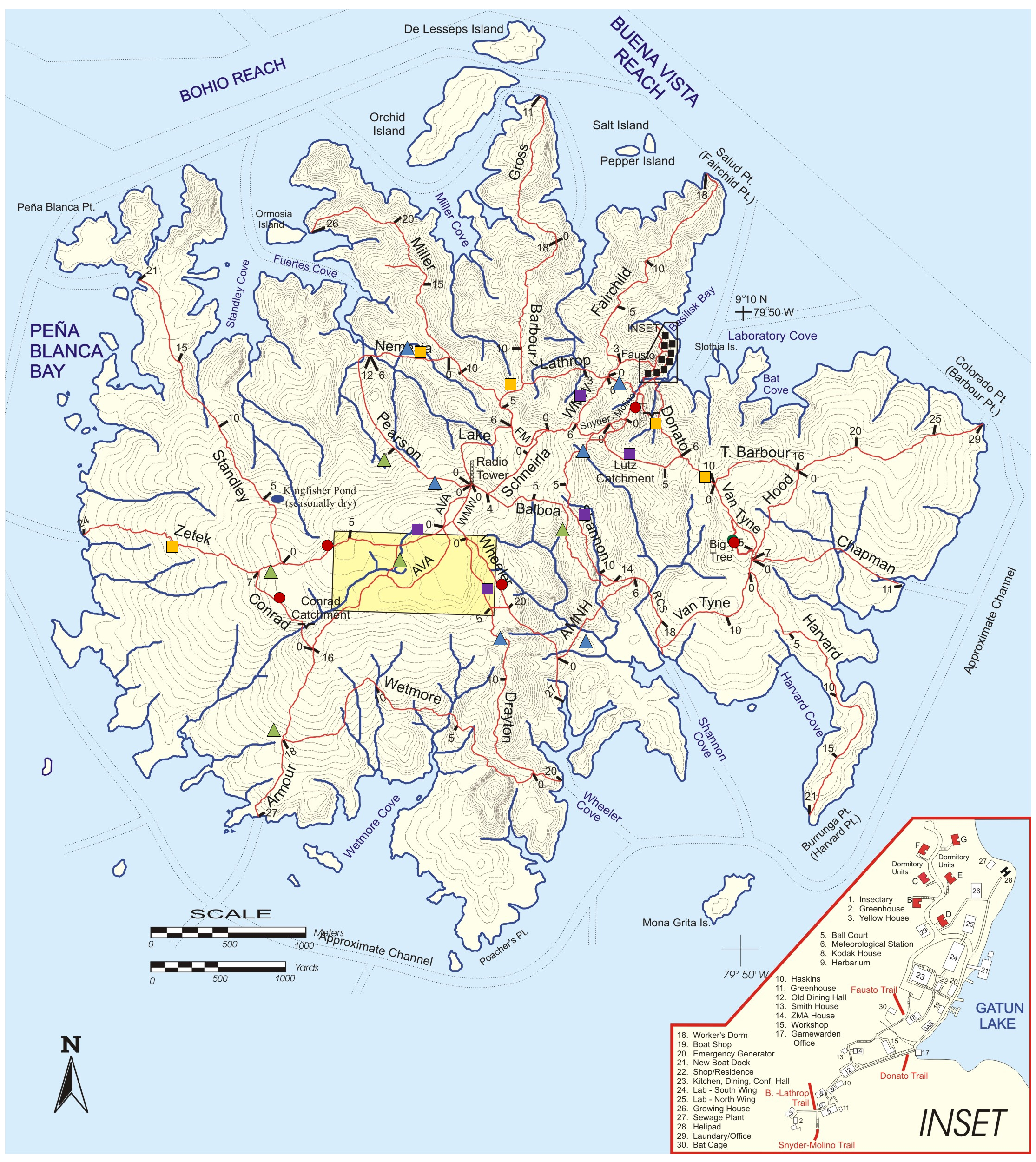


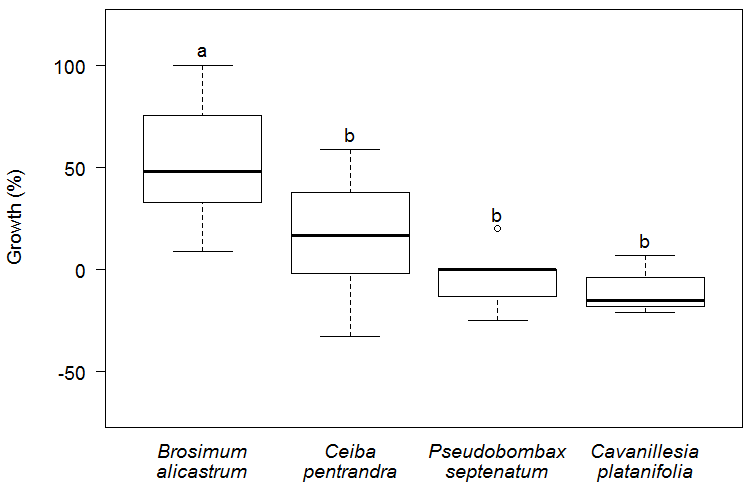


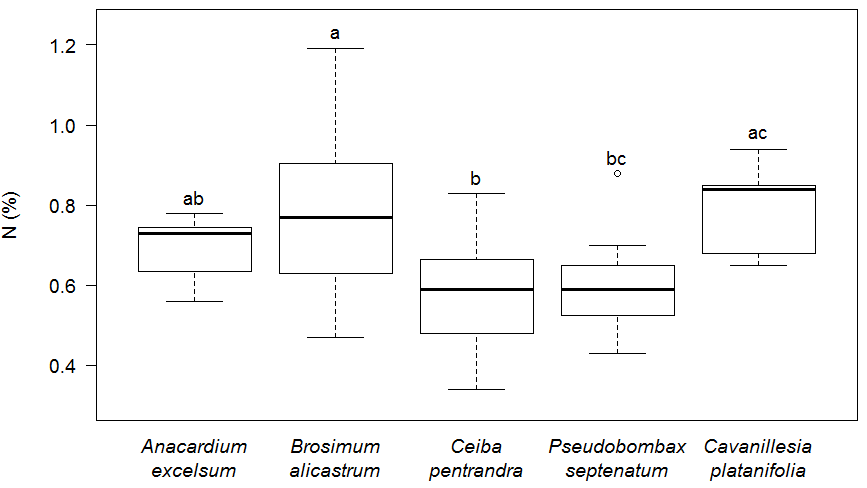

Supplement: Additional Information [file supp_plu073_plu073supp.docx]
