# Supplementary material for: Host tree phenology affects vascular epiphytes at the physiological, demographic and community level
Source: AoB Plants. 2014 Nov 11;7:plu073. doi: 10.1093/aobpla/plu073 (PMC4287691; doi:10.1093/aobpla/plu073)
Supplement: Additional Information [file supp_plu073_plu073supp_table1.docx]

**Table S1.** **Species abundances of epiphytes and hemiepiphytes.** The studied species were ***Ana****cardium excelsum* (n = 5), ***Bro****simum alicastrum* (n = 4), ***Cei****ba pentandra* (n = 5), ***Pse****udobombax septenatum* (n = 5) and ***Cav****anillesia platanifolia* (n = 5). Nutrient concentrations are usually averages of six replicates, exceptional cases with a single sample are shown in italics. Some δ^13^C values were taken from other publications ([^1^ Zotz and Ziegler, 1997](#_ENREF_3), [² Zotz, 2004](#_ENREF_1)). δ^13^C and δ^15^N in ‰, C and N in %. * Hemiepiphyte or nomadic vine sensu [Zotz (2013)](#_ENREF_2). † Nomadic vines with established ground contact via roots (excluded from further analyses).

| **Taxon** | **δ^13^C** | **δ^15^N** | **C** | **N** | **Ana** | **Bro** | **Cei** | **Pse** | **Cav** |
| --- | --- | --- | --- | --- | --- | --- | --- | --- | --- |
| Araceae |  |  |  |  |  |  |  |  |  |
| *Anthurium acutangulum* Engl. | -28.5^1^ | — | — | — | 0 | 5 | 0 | 0 | 0 |
| *Anthurium brownii* Mast. | -30.6 | 0.3 | 48.8 | 1.2 | 22 | 47 | 77 | 1 | 0 |
| *Anthurium clavigerum* Poepp.† | -26.0^2^ | — | — | — | 4 | 4 | 0 | 0 | 0 |
| *Anthurium durandii* Engl. | -27.7 | -1.4 | 45.7 | 0.9 | 22 | 82 | 12 | 0 | 1 |
| *Anthurium friedrichsthalii* Schott | -28.4 | 0.2 | 43.9 | 1.1 | 6 | 107 | 4 | 0 | 1 |
| *Anthurium gracile* (Rudge) Lindl. | -28.9^1^ | — | — | — | 4 | 0 | 0 | 0 | 0 |
| *Anthurium schlechtendalii* Kunth | -29.5 | -1.3 | 43.2 | 1.3 | 13 | 1 | 0 | 0 | 0 |
| *Monstera adansonii* Schott† | *-28.3* | *5.8* | *42.9* | *2.5* | 0 | 0 | 3 | 1 | 0 |
| *Monstera dilacerata* (K. Koch &Sello) K. Koch† | -27.7^1^ | — | — | — | 0 | 0 | 1 | 0 | 0 |
| *Monstera dubia* (Kunth) Engl. & K. Krause† | *-32.1* | *4.0* | *42.8* | *3.5* | 1 | 1 | 6 | 0 | 0 |
| *Philodendron inaequilaterum* Liebm.† | — | — | — | — | 0 | 0 | 1 | 0 | 0 |
| *Philodendron radiatum* Schott | -30.8 | 0.0 | 48.0 | 2.4 | 55 | 20 | 9 | 1 | 0 |
| *Philodendron sagittifolium* Liebm.* | *-31.1* | *-2.1* | *40.6* | *1.1* | 36 | 0 | 13 | 0 | 0 |
| *Philodendron tripartitum* (Jacq.) Schott | *-31.3* | *2.9* | *41.5* | *2.5* | 2 | 1 | 4 | 2 | 0 |
| *Stenospermation angustifolium* Hemsl. | -30.3 | -1.6 | 40.9 | 1.3 | 1335 | 20 | 120 | 2 | 0 |
| *Syngonium erythrophyllum* Birdsey ex G.S.* | *-28.6* | *-1.0* | *45.4* | *2.1* | 4 | 0 | 0 | 2 | 0 |
| Aspleniaceae |  |  |  |  |  |  |  |  |  |
| *Asplenium auritum* Sw. | -31.8 | -1.6 | 43.2 | 1.4 | 550 | 0 | 0 | 0 | 0 |
| *Asplenium serratum* L. | -32.1 | -1.0 | 40.9 | 2.2 | 4 | 5 | 5 | 0 | 0 |
| Bromeliaceae |  |  |  |  |  |  |  |  |  |
| *Aechmea setigera* Mart. ex Schult. &Schult.f. | *-13.1* | *-2.7* | *45.0* | *0.5* | 0 | 9 | 4 | 1 | 0 |
| *Aechmea tillandsioides* (Mart. ex Schult. & Schult. f.) Baker | -17.2^2^ | — | — | — | 1 | 0 | 0 | 0 | 0 |
| *Guzmania lingulata* (L.) Mez | -34.1^1^ | — | — | — | 25 | 0 | 0 | 0 | 0 |
| *Guzmania monostachia* (L.) Rusby ex Mez | -27.5^1^ | — | — | — | 4 | 0 | 0 | 0 | 0 |
| *Tillandsia anceps* G. Lodd. | -30.1 | -2.9 | 45.6 | 0.6 | 925 | 0 | 0 | 0 | 0 |
| *Tillandsia bulbosa* Hook. | *-14.0* | *0.8* | *45.7* | *0.6* | 49 | 192 | 149 | 0 | 0 |
| *Tillandsia monadelpha* (E. Morren) Baker | -26.7^1^ | — | — | — | 7 | 0 | 0 | 0 | 0 |
| *Werauhia gladioliflora* (H. Wendl.) J.R. Grant | -28.6^2^ | — | — | — | 30 | 0 | 0 | 0 | 0 |
| *Werauhia sanguinolenta* Cogn. & Marchal) J.R. Grant | -33.2^1^ | — | — | — | 0 | 0 | 1 | 1 | 0 |
| Cactaceae |  |  |  |  |  |  |  |  |  |
| *Epiphyllum phyllantus* L. | -14.7 | 3.5 | 45.6 | 0.7 | 5 | 48 | 16 | 0 | 0 |
| *Rhipsalis cassytha* Gaertn. | *-15.2* | *2.2* | *46.9* | *0.9* | 0 | 5 | 10 | 0 | 0 |
| Clusiaceae |  |  |  |  |  |  |  |  |  |
| *Clusia uvitana* Pittier* | *-21.2* | *1.3* | *45.3* | *0.8* | 19 | 23 | 76 | 0 | 0 |
| Cyclanthaceae |  |  |  |  |  |  |  |  |  |
| *Ludovia integrifolia* (Woodson) Harling | -31.4 | -3.2 | 41.8 | 1.5 | 8 | 0 | 210 | 0 | 0 |
| Davalliaceae |  |  |  |  |  |  |  |  |  |
| *Nephrolepis pendula* (Raddi) J. Sm. | -29.0 | -1.8 | 44.7 | 1.2 | 139 | 23 | 217 | 0 | 0 |
| Dryopteridaceae |  |  |  |  |  |  |  |  |  |
| *Elaphoglossum* cf *doanense* L.D. Gómez | -30.3 | -2.4 | 42.5 | 0.8 | 234 | 0 | 0 | 0 | 0 |
| *Elaphoglossum hayesii* (Mett. Ex Kuhn) Maxon | -31.9 | -2.8 | 43.7 | 0.9 | 334 | 0 | 0 | 0 | 0 |
| *Elaphoglossum herminieri* (Bory& Fee) Moore | -29.2 | -4.5 | 43.1 | 0.7 | 905 | 0 | 205 | 0 | 0 |
| *Elaphoglossum latum* (Mickel) Atehortúa ex Mickel | -29.5 | -3.1 | 40.0 | 0.8 | 4 | 0 | 0 | 0 | 0 |
| *Elaphoglossum peltatum* (Sw.) Urb. | -32.5 | -3.1 | 43.9 | 1.4 | 140 | 0 | 0 | 0 | 0 |
| *Elaphoglossum sporadolepis* (Kunze) Moore | -30.4 | -2.9 | 42.2 | 0.8 | 210 | 2 | 100 | 1 | 0 |
| Gesneriaceae |  |  |  |  |  |  |  |  |  |
| *Codonanthe crassifolia* (H. Focke) C.V. Morton | -28.2 | 0.5 | 41.5 | 1.3 | 0 | 377 | 0 | 0 | 0 |
| Hymenophyllaceae |  |  |  |  |  |  |  |  |  |
| *Hymenophyllum brevifrons* Kunze | -31.1 | -0.9 | 49.8 | 1.6 | 6 | 0 | 0 | 0 | 0 |
| *Trichomanes* cf *punctatum* W. Boer | *-21.6* | *-1.9* | *41.8* | *3.0* | 24 | 0 | 0 | 0 | 0 |
| Lycopodiaceae |  |  |  |  |  |  |  |  |  |
| *Huperzia dichotoma* (Jacq.) Trevis | -29.5^2^ | — | — | — | 5 | 5 | 4 | 0 | 0 |
| Orchidaceae |  |  |  |  |  |  |  |  |  |
| *Aspasia principissa* Rchb. f. | -31.5 | -0.6 | 48.8 | 1.4 | 78 | 5 | 39 | 0 | 0 |
| *Brassia caudata* (L.) Lindl. | -27.2^1^ | — | — | — | 1 | 0 | 0 | 0 | 0 |
| *Catasetum viridiflavum* Hook. | -29.3^1^ | — | — | — | 0 | 5 | 10 | 2 | 0 |
| *Cattleya patinii* Cogn. | *-15.9* | *0.3* | *49.8* | *0.7* | 0 | 5 | 20 | 0 | 0 |
| *Caularthron bilamellatum* (Reichb. f.) Schult. | -17.9 | -2.0 | 47.6 | 1.0 | 4 | 9 | 94 | 369 | 31 |
| *Dichaea panamensis* Lindl. | -30.4 | -2.2 | 45.2 | 1.6 | 168 | 64 | 0 | 0 | 0 |
| *Dimerandra emarginata* (G. Mey) Hoehne | -27.6 | -2.6 | 46.4 | 1.1 | 512 | 259 | 1284 | 1506 | 1 |
| *Elleanthus longibracteatus* (Lindl. ex Griseb.) Fawc. | -29.0 | 0.4 | 50.2 | 0.9 | 4 | 0 | 352 | 0 | 0 |
| *Encyclia chacaoensis* (Rchb. f.) Dressler & G.E. Pollard | -29.1^1^ | — | — | — | 0 | 0 | 20 | 2 | 0 |
| *Encyclia* cf *chimborazoensis* (Schlechter) Dressl. | -27.8^1^ | — | — | — | 110 | 0 | 0 | 0 | 0 |
| *Epidendrum nocturnum* Jacq. | -25.6^1^ | — | — | — | 6 | 0 | 0 | 12 | 0 |
| *Epidendrum rigidum* Jaqc. | *-16.8* | *-4.6* | *47.7* | *0.6* | 5 | 5 | 40 | 0 | 0 |
| *Epidendrum schlechterianum* Ames | -15.6 | -2.0 | 47.9 | 0.8 | 0 | 30 | 28 | 0 | 0 |
| *Leucohyle subulata* ((Sw.) Schltr.) | -17.1 | 3.1 | 44.7 | 1.5 | 0 | 2 | 0 | 0 | 0 |
| *Lockhartia acuta* (Lindl.) Reichb. f. | -20.1^1^ | — | — | — | 8 | 0 | 0 | 0 | 0 |
| *Maxillaria acervata* Rchb. f. | -31.6 | -1.3 | 52.1 | 1.3 | 227 | 0 | 0 | 0 | 0 |
| *Maxillaria camaridii* Reichb. f. | -28.2^1^ | — | — | — | 0 | 0 | 12 | 40 | 0 |
| *Maxillaria crassifolia* (Lindl.) Rchb. | -13.4^1^ | — | — | — | 53 | 0 | 0 | 0 | 0 |
| *Maxillaria uncata* Lindl. | -28.2 | -1.6 | 45.4 | 0.8 | 284 | 651 | 1454 | 0 | 0 |
| *Maxillaria variabilis* Bateman ex Lind. | -32.2^1^ | — | — | — | 36 | 0 | 0 | 0 | 0 |
| *Oncidium ampliatum* Lindl. | -15.3 | -1.5 | 46.6 | 1.1 | 0 | 0 | 36 | 4 | 0 |
| *Oncidium stipitatum* Lindl. ex Benth. | -14.5^1^ | — | — | — | 0 | 5 | 4 | 0 | 0 |
| *Pleurothallis brighamii* S. Wats. | -28.0 | -1.6 | 44.6 | 0.8 | 1583 | 476 | 358 | 0 | 0 |
| *Pleurothallis grobyi* Bateman ex Lindl. | -30.0^2^ | — | — | — | 0 | 10 | 0 | 0 | 0 |
| *Pleurothallis verecunda* Schlechter | -14.0^1^ | — | — | — | 0 | 9 | 0 | 0 | 0 |
| *Polystachya foliosa* (Lindl.) Reichb. f. | -27.9 | -1.3 | 44.4 | 1.4 | 13 | 33 | 145 | 11 | 0 |
| *Scaphyglottis behrii* (Rchb. f.) Benth. & Hook. f. ex Hemsl. | -29.7 | -1.2 | 47.2 | 1.0 | 1527 | 68 | 20 | 24 | 0 |
| *Scaphyglottis longicaulis* S. Wats. | *-31.5* | *-1.5* | *46.2* | *1.1* | 319 | 0 | 60 | 0 | 0 |
| *Sobralia fenzliana* Rchb. f. | -29.3 | -0.2 | 50.6 | 1.7 | 46 | 6 | 32 | 0 | 0 |
| *Sobralia fragrans* Lindl. | *-30.5* | *-0.5* | *51.4* | *1.2* | 51 | 10 | 38 | 0 | 0 |
| *Sobralia suaveolens* Rchb. f. | -29.8^1^ | — | — | — | 0 | 0 | 0 | 12 | 0 |
| *Stelis crescentiicola* Schltr. | -29.2 | -1.1 | 47.5 | 0.8 | 0 | 0 | 1364 | 0 | 0 |
| *Trichopilia maculata* Rchb. f. | -29.3 | -2.2 | 46.9 | 1.1 | 52 | 49 | 0 | 0 | 0 |
| *Trigonidium egertorianum* Bateman ex Lindl. | -30.8^1^ | — | — | — | 40 | 0 | 76 | 0 | 0 |
| *Xylobium foveatum* (Lindl.) G. Nicholson | *-30.2* | *-0.3* | *50.3* | *1.3* | 1 | 0 | 0 | 0 | 0 |
| Piperaceae |  |  |  |  |  |  |  |  |  |
| *Peperomia glabella* (Sw.) A. Dietr. | *-31.8* | *-4.1* | *44.0* | *0.9* | 0 | 5 | 0 | 0 | 0 |
| *Peperomia macrostachya* (Vahl) A. Dietr. | -29.6 | -0.2 | 43.6 | 1.5 | 0 | 21 | 0 | 0 | 3 |
| *Peperomia obscurifolia* C. DC. | -30.2 | -2.1 | 43.1 | 1.3 | 111 | 21 | 315 | 0 | 0 |
| *Peperomia obtusifolia* (L.) A. Dietr. | -27.4 | -1.9 | 44.6 | 1.3 | 66 | 0 | 48 | 0 | 0 |
| *Peperomia rotundifolia* (L.) H.B.K. | -35.0^1^ | — | — | — | 0 | 40 | 0 | 0 | 0 |
| Polypodiaceae |  |  |  |  |  |  |  |  |  |
| *Campyloneurum aphanophlebium* (Kunze) T. Moore | *-32.9* | *-2.9* | *46.7* | *0.9* | 5 | 0 | 0 | 0 | 0 |
| *Campyloneurum phyllitidis* (L.) K. Presl. | -30.5 | -1.2 | 46.1 | 1.3 | 88 | 20 | 66 | 0 | 0 |
| *Microgramma lycopodioides* (L.) Copel. | -23.1^2^ | — | — | — | 0 | 5 | 0 | 0 | 0 |
| *Niphidium crassifolium* (L.) Lellinger | -27.2 | -2.0 | 43.5 | 0.8 | 134 | 495 | 1242 | 282 | 37 |
| *Pecluma pectinata* (L.) M.G. Price | -30.9 | -0.5 | 47.7 | 1.7 | 80 | 15 | 24 | 0 | 0 |
| *Polypodium percussum* Cav. | -28.2^1^ | — | — | — | 18 | 5 | 42 | 0 | 0 |
| *Polypodium polypodioides* (L.) Watt | -29.8 | 0.6 | 45.1 | 1.3 | 0 | 20 | 190 | 9 | 1 |
| Rubiaceae |  |  |  |  |  |  |  |  |  |
| *Cosmibuena skinneri* (Oerst.) Hemsl.* | -28.3 | -3.7 | 42.0 | 0.7 | 99 | 5 | 28 | 0 | 0 |
| Vittariaceae |  |  |  |  |  |  |  |  |  |
| *Ananthocorus angustifolius* (Sw.) Und. & Max. | -31.2^1^ | — | — | — | 41 | 0 | 30 | 0 | 0 |
| *Vittaria lineata* (L.) J. Sm. | -30.2 | -0.1 | 39.7 | 0.9 | 61 | 102 | 109 | 13 | 0 |

**LITERATURE CITED**

Zotz G. 2004. How prevalent is crassulacean acid metabolism among vascular epiphytes? *Oecologia* 138: 184-192.

Zotz G. 2013. Hemiepiphyte: a confusing term and its history. *Annals of Botany* 111: 1015-1020.

Zotz G, Ziegler H. 1997. The occurrence of crassulacean acid metabolism among vascular epiphytes from Central Panama. *New Phytologist* 137: 223-229.
