# Supplementary material for: Host tree phenology affects vascular epiphytes at the physiological, demographic and community level
Source: AoB Plants. 2014 Nov 11;7:plu073. doi: 10.1093/aobpla/plu073 (PMC4287691; doi:10.1093/aobpla/plu073)
Supplement: Additional Information [file supp_plu073_plu073supp_table2.docx]

**Table S2.** **Summary of ANCOVA results for size dependence of four leaf traits.** Analysed were size and its significant interaction (*) with ‘host species’ of the studied leaf traits (SLA, δ^13^C, δ^15^N, N) for the focal epiphyte species (**all** studied host species, ***Ana****cardium excelsum*, ***Bro****simum alicastrum*, ***Cei****ba pentandra*, ***Pse****udobombax septenatum*, ***Cav****anillesia platanifolia*, other = all other except the specified). ↑ Positive correlation, ↓ negative correlation, – no correlation.

|  | **SLA** | **δ^13^C** | **δ^15^N** | **N** |
| --- | --- | --- | --- | --- |
| ***Dimerandra emarginata*** | *↓all  p < 0.001  F_(2,60)_ = 22.4 | No size dependence | *– Pse ↓Bro/Cei  p < 0.05  F_(2,60)_ = 3.3 | No size dependence |
| ***Maxillaria uncata*** | No size dependence | ↓all  p < 0.05  F_(1,36)_ = 6.2 | ↓all  p < 0.001  F_(1,32)_ = 14.3 | No size dependence |
| ***Niphidium crassifolium*** | ↓all  p < 0.05  F_(1,73)_ = 5.4 | *– other ↓Cav  p < 0.05  F_(4,74)_ = 4 | *↑other ↓Ana  p < 0.05  F_(4,70)_ = 3.2 | No size dependence |
| ***Scaphyglottis behrii*** | *↑Bro ↓other  p < 0.05  F_(3,20)_ = 4.5 | ↑all  p < 0.05  F_(1,22)_ = 15.2 | No size dependence | No size dependence |
| ***Vittaria lineata*** | *↑other ↓Bro  p < 0.05  F_(2,6)_ = 11.3 | No size dependence | No size dependence | No size dependence |
